# Pharmacokinetics and Metabolite Profiling of Trepibutone in Rats Using Ultra-High Performance Liquid Chromatography Combined With Hybrid Quadrupole-Orbitrap and Triple Quadrupole Mass Spectrometers

**DOI:** 10.3389/fphar.2019.01266

**Published:** 2019-11-04

**Authors:** Zhi Sun, Jie Yang, Liwei Liu, Yanyan Xu, Lin Zhou, Qingquan Jia, Yingying Shi, Xiangyu Du, Jian Kang, Lihua Zuo

**Affiliations:** ^1^Department of Pharmacy, The First Affiliated Hospital of Zhengzhou University, Zhengzhou, China; ^2^Henan Engineering Research Center of Clinical Mass Spectrometry for Precision Medicine, Zhengzhou, China; ^3 ^School of Pharmacy, Shenyang Pharmaceutical University, Shenyang, China; ^4^Department of Basic Medical Sciences, Henan University, Kaifeng, China

**Keywords:** trepibutone, pharmacokinetics, metabolite profiling, UHPLC-MS/MS, UHPLC-Q-orbitrap HRMS

## Abstract

Trepibutone was widely used for cholelithiasis, cholecystitis, biliary tract dyskinesia, cholecystectomy syndrome, and chronic pancreatitis in clinic. However, few investigations on trepibutone have been conducted. In this study, an accurate, sensitive, and selective analytical method was developed and successfully applied to assess the pharmacokinetic behavior of trepibutone in rats. Trepibutone and carbamazepine (internal standard, IS) were quantified using multiple reaction monitoring (MRM) mode with the transitions of *m/z* 311.09→265.08 and *m/z* 237.06→194.08, respectively. The linearity, precision, accuracy, extraction recovery, matrix effect, and stability of the established method were all excellent within acceptable range. A total of 30 metabolites were identified in plasma and urine by Q-Exactive high resolution mass spectrometry, and several common metabolic pathways were observed such as dealkylation, oxidation, reduction, glucuronidation, and so on. This research provides more information on trepibutone in pharmacodynamics and toxicology and will assist the usage of trepibutone in clinical.

## Introduction

Trepibutone (4-oxo-4-(2,4,5-triethoxyphenyl) butanoic acid), a remarkable biliary smooth muscle relaxant without an anticholinergic effect, has been widely used for the treatment of cholelithiasis, cholecystitis, biliary tract dyskinesia, cholecystectomy syndrome, and chronic pancreatitis. However, few researchers have payed attention to the metabolic process of trepibutone.

The pharmacokinetic study exhibits the characteristic of drugs *in vivo* (Ren et al., 2014;Yuan et al., 2015). The pharmacokinetic parameters possess a practical significance in clinical rational drug use (Song et al., 2018; Tate et al., 2018) and these parameters can assist researchers in discovering interesting scientific problems (Huang et al., 2016;Karrer et al., 2018). However, lack of information limits researchers’ understanding of the pharmacokinetic behavior of trepibutone. The study of drug metabolism plays a pivotal role in the drug discovery process (Ezzeldin et al., 2017;Godinho et al., 2018;Shankar et al., 2018), which is necessary to explore the metabolic fate thoroughly. The information regarding case reports associated with biotransformation and identification of metabolites will provide researchers with a better understanding about the activity and risks of trepibutone (Huskey et al., 2016; Iegre et al., 2016; Aras et al., 2017; Glaenzel et al., 2018; He and Wan, 2018; Mizuno et al., 2018). Hence, explicit metabolite profiles and metabolic pathways are essential for further research (Isin et al., 2012; Pozo et al., 2015; Xing et al., 2017).

A sensitive and effective ultra-high performance liquid chromatography with tandem mass spectrometry (UHPLC-MS/MS) method in multiple reaction monitoring (MRM) mode was developed and validated in order to study the pharmacokinetic properties of trepibutone. The present results provide valuable information for better understanding the physiological disposition and pharmacological mechanism of trepibutone as well as its potential clinical significance to treatment. For the metabolite detection of trepibutone *in vivo*, a UHPLC-Q-orbitrap HRMS method with data-dependent MS^2^ capture mode was established. As a result, 30 metabolites in total were identified in plasma and urine. To the best of our knowledge, it is the first time such studies have been to carried out on trepibutone. These results could provide helpful information for the clinical application and pharmacological action mechanism of trepibutone, and they might also lay a preliminary scientific foundation for the further research.

## Materials and Methods

### Chemicals, Materials and Animals

Trepibutone and carbamazepine (internal standard, IS) were purchased from National Institute for the Control of Pharmaceutical and Biological Products (Beijing, China) and the purity was more than 98%. Acetonitrile and methanol were HPLC grade and obtained from Fisher Scientific (Fair Lawn, NJ, USA). Formic acid of HPLC grade was purchased from the Aladdin Industrial Corporation (Shanghai, China). All the other reagents were analytical grade. Deionized water was prepared *via* a Milli-Q water purification system (Millipore, Milford, MA, USA).

Sprague-Dawley (SD) rats, weighing 200 ± 20 g, were acquired from the Experimental Animal Center of Zhengzhou University (Zhengzhou, China). In order to adapt to the environment, all animals were kept in an environment where temperature (20 ± 2°C), humidity (60 ± 5%), and light (12 h light/12 h dark cycle) were controlled for one week before the experiment. All protocols of animal experiments were in accordance with the Regulations of Experimental Animal Administration issued by the Animal Ethics Committee of the institution of First Affiliated Hospital of Zhengzhou University.

### UHPLC-MS/MS Instrument and Conditions for Pharmacokinetic Study

Chromatography separation was carried on the UHPLC Dionex Ultimate 3000 system (Thermo Scientific, San Jose, USA) and a Waters ACQUITY UPLC C_18_ column (2.1 mm × 50 mm, 1.7 μm) was applied at temperature of 40 °C. The mobile phases included 0.1% formic acid in water (A) and acetonitrile (B) with gradient elution, and the procedure was as follows: 0∼1.0min, 5% B; 1.0∼5.0 min, 5%∼100% B; 5.0∼8.0 min, 100% B; 8.0∼8.1 min, 100%∼5% B; 8.1∼10.0 min, 5% B. The auto-sampler was conditioned at 10°C and the flow rate was set at 0.20 ml·min^-1^ with the injection volume 3 μl.

A TSQ-ALtis Triple series quadrupole mass spectrometer (Thermo Scientific, San Jose, USA) equipped with a heated electrospray ionization (HESI) source was used for data acquisition and analysis. The optimized instrument parameters were shown as below: the spray voltage: + 3.5 kV for positive mode or - 2.5 kV for negative mode; the sheath gas pressure: 50 arb; the aux gas pressure: 10 arb; the sweep gas pressure: 0 arb; Ion Transfer Tube Temp (°C): 325 °C; Vaporizer Temp (°C): 350 °C; Cycle Time(sec): 0.8 s; Q1 Resolution (FWHM): 0.7; Q3 Resolution (FWHM): 1.2; CID Gas (mTorr): 2; and Chromatographic Peak Width (sec): 6 s. Besides, Thermo Trace Finder 4.1 General Quan (Thermo Scientific, San Jose, USA) was used to process the acquired data.

### UHPLC-Q-Orbitrap HRMS Instrument and Conditions for Metabolite Profiling

Chromatographic separation was performed on UHPLC Dionex Ultimate 3000 system (Thermo Scientific, San Jose, USA) using a Waters ACQUITY UPLC^®^ HSS T3 column (2.1 mm × 100 mm, 1.7 µm) and the column temperature was set at 40 °C. The gradient elution was achieved in water (A, 0.1% formic acid) and acetonitrile (B) as mobile phases, and the procedure was as follows: 0.0∼3.0 min, 5.0% A; 3.0∼16.0 min, 5.0%∼15.0% A; 16.0∼20.0 min, 15.0%∼22.0% A; 20.0∼26.0 min, 22.0%∼55.0% A; 26.0∼30.0 min, 55.0%∼100.0% A; 30.0∼35.0 min, and 100% A at a flow of 0.2 ml·min^-1^. The auto-sampler was maintained at 10 °C and the injection volume was 5 μl for analysis.

A Q Exactive high resolution mass spectrometry equipped with an electrospray ionization (ESI) ion source was connected to the UHPLC system. Parameters such as the temperatures of auxiliary gas, ion source and capillary were set at 300 °C, 350 °C, and 320 °C, respectively, with the flow rate of the auxiliary gas at 10 μl·min^-1^. The gradient collision energy was set at 20, 30, and 40 eV. The spray voltage was set at 3.50 kV and 2.80 kV with the sheath gas flow rate at 40 μl·min^-1^ and 38 μl·min^-1^ for the positive mode and negative mode, respectively. Samples were detected through acquisition mode of Full scan/dd MS^2^ with a range of 80 ∼ 1200 *m/z* at the mass resolution of 17,500 in MS/MS.

### Sample Preparation for Metabolite Identification

The SD rats, which were fasted but drank freely, were kept in metabolic cages individually to collect blood, urine, and faeces samples. Blood samples were collected through orbital vein for 0.5 ml each time before administration and at 10 min, 30 min, 1 h, 2 h, and 4 h after administration. Samples in heparinized were centrifuged at 5,000 rpm for 10 min to obtain plasma. The urine and faeces samples were collected from metabolic cages at the same time.

The sample processing was as follows: protein precipitation was processed by adding acetonitrile (300 μl) to rat plasma sample (100 μl) and then centrifuged at 13,000 rpm for 10 min after vortexing for 3 min. The supernatant was transferred into a centrifuge tube and evaporated to dryness in a vacuum centrifugal concentrator. The residue was dissolved in 50 μl of 50% methanol with vortex-mixing for 1 min. The centrifugation process was carried out for 5 min and 5 μl supernatant was used for analysis.

The urine sample (200 μl) was spiked with 200 μl pure water, vortexing for 1 min. After centrifuging at 13,000 rpm for 10 min, the supernatant was filtered by 0.22 µm microporous filter membrane. The faeces sample (0.2 g) was weighed precisely, and, after adding 2 ml methanol and ultrasonic dissolving, the supernatant was transferred and centrifuged as the above. Then, the supernatant was filtered by 0.22 µm microporous filter membrane into a sample bottle before analysis.

### Preparation of Calibration Standard and Quality Control Samples

The primary stock solutions of trepibutone and IS used as calibration standards were prepared in methanol at a concentration of 1.0 mg·ml^-1^. The working solutions for the standard curve were acquired from stock solutions, which were serially diluted to the desired concentration with methanol. The final concentration of IS solution was 50 ng·ml^-1^ in methanol. Calibration standard samples were prepared with 90 μl blank rat plasma and 10 μl working solutions spiked with the concentration ranges within 1∼1000 ng·ml^-1^. The quality control (QC) samples at low, medium, and high concentration were prepared as the above way with three concentration at 3.0, 400, and 800 ng·ml^-1^.

### Sample Preparation for Pharmacokinetic Study

The extractions of trepibutone and IS were conducted with a one-step protein precipitation method. Plasma samples (100 μl) were spiked with 10 μl of the IS working solution, and then the protein precipitation of mixture was carried out by adding 300 μl of acetonitrile. The tubes were then vortex-mixed for 3.0 min and centrifuged at 13,000 rpm for 10 min. The supernatant (100 μl) was then transferred into a sample bottle and 5 μl was used for analysis.

### Method Validation

The validation of the bioanalytical methods was in accordance with FDA guidance, which contained the contents of linearity, matrix effect, accuracy, precision, and stability (U.S. Food and Drug Administration, 2013).

#### Linearity and Lower Limit of Quantification (LLOQ)

The *1/x* *^2^* weighted least-squares linear regression model was applied to fit the calibration curve, which was constructed by plotting the peak-area ratio of trepibutone to IS versus the concentrations of the analyte. The concentrations of analyte were 1, 2, 10, 50, 200, 500, and 1000 ng·ml^-1^, respectively. The linearity of a calibration curve was evaluated *via* a correlation coefficient value (*r*) and the acceptance criterion was more than 0.99. The lower limit of quantitation (LLOQ) was at least 10 times of signal-to-noise ratio and the precision (expressed by relative standard deviation, RSD) and accuracy (calculated by relative error, RE) at LLOQ should have been within ±20%.

#### Precision and Accuracy

The intra-day precision and accuracy of the developed method were evaluated through six replicates of LLOQ samples at 1 ng·ml^-1^ and QC samples at 3.0, 400, and 800 ng·ml^-1^ on the same day. The inter-day accuracy and precision were studied by LLOQ samples and three levels of QC samples on three consecutive days. The precision and accuracy were expressed by RSD and RE, respectively.

#### Extraction Recovery and Matrix Effect

Extraction recovery of the analyte was evaluated by comparing peak areas of analyte spiked with the plasma before and after extraction at three QC levels and at one concentration for the IS. The matrix effect was evaluated using extracted blank samples spiked with trepibutone at three QC concentrations by comparing peak area of analyte in post-exacted spiked samples with that of a corresponding neat solution. An endogenous matrix effect was suggested if the ratio was not within the range 85%–115%.

#### Stability

Six replicates of plasma samples were used to assay the stability at three QC levels and under different conditions: exposure at room temperature for 4 h, three freeze/thaws cycles, storage at 4 °C for 24 h, and at −20 °C for 14 days. The post-preparation stability was conducted by analyzing the extracted QC samples kept in the auto-sampler for 24 h. If the accuracy and precision were within the acceptable limits ( ± 15% RE and ≤15% RSD), samples were regarded as stable.

#### Dilution Test

Dilution test was used to assess the validity of the dilutions, which could prove that the concentrations above upper limits of quantification (ULOQ) that may have been present in samples were accurately measured. The dilution was tested by analyzing six replicates of trepibutone at 30-fold dilution to evaluate the dilution effect on the accuracy and precision of the experiment. If the mean accuracy and precision were within ± 15% of the nominal concentration, the dilution integrity was considered to be verified.

### Pharmacokinetic Study

Sprague-Dawley rats were divided into three groups randomly and fasted for 12 h, but they were given free access to water before experiments. Each group (n = 6) was given trepibutone orally at a dose of 4.2, 8.4, and 12.6 mg·kg^-1^, respectively. Blood samples (200 μl) were collected at 0, 3, 15, 30, 45 min, 1, 2, 4, 8, 12, 18, and 24 h after dosing. The blood samples were immediately centrifuged at 4,000 rpm for 10 min and stored at -20 °C until analysis.

### Data Processing and Analysis

The maximum plasma concentration (*C* *_max_*) and the time of the maximum plasma concentration (*T* *_ma_*_x_) were obtained directly from drug concentration-time curve based on measured data. The other pharmacokinetic parameters were calculated *via* DAS 2.0 software (Chinese Pharmacological Society), such as *t* *_1/2_* (the elimination half-life), *AUC* *_0-t_* (the area under the plasma concentration–time curve to the last measurable plasma time concentration), and *AUC* *_0-∞_* (the area under the plasma concentration–time curve to time infinity). Next, these pharmacokinetic parameters were analyzed through the SPSS software.

The HRMS data of all tests and control samples were processed in the Compound Discoverer 2.1 (CD, Thermo Fisher Scientific) to extract the metabolite-related dataset. The extracted data included molecular formula, molecular weight, and biotransformation, which assisted in conjecturing structure. Then, the speculative structure and mass spectrometry were further exported to the Mass Frontier 7.0 (MF, Thermo Fisher Scientific) to observe whether the structure and fragments matched.

## Results and Discussion

### Method Development

#### Optimization of the Sample Preparation and Chromatographic Condition

Protein precipitation was used as the sample preparation method ultimately due to the simple process and desired recovery of the analyte. Carbamazepine was chosen as the IS considering its excellent stability and suitable retention. In addition, the ionization and extraction efficiency of carbamazepine was similar to trepibutone.

In order to obtain higher responses, suitable retention behaviors and symmetrical peak shapes for the analyte, various mobile phase conditions (methanol, acetonitrile, different proportions of formic acid, ammonium acetate, and 100% water) were tested as potential mobile phases. Finally, acetonitrile-water containing 0.1% formic acid was chosen for its good peak shapes. It was also noted that gradient elution could dramatically narrow the peak shape and improve the response intensity and resolution of the analyte and IS.

#### Optimization of Mass Spectrometry

To maximize the MS responses to trepibutone and IS, some instrument parameters were investigated and optimized, among which the optimized collision energy of analyte was 18.11 V, and the optimized collision energy of IS was 19.48 V, respectively. The precursor-to-product ion transitions of *m/z* 311.09 → 265.08 for trepibutone and *m/z* 237.06 → 194.08 for IS were selected under MRM mode.

### Method Validation

#### Selectivity

**Figure 1:**
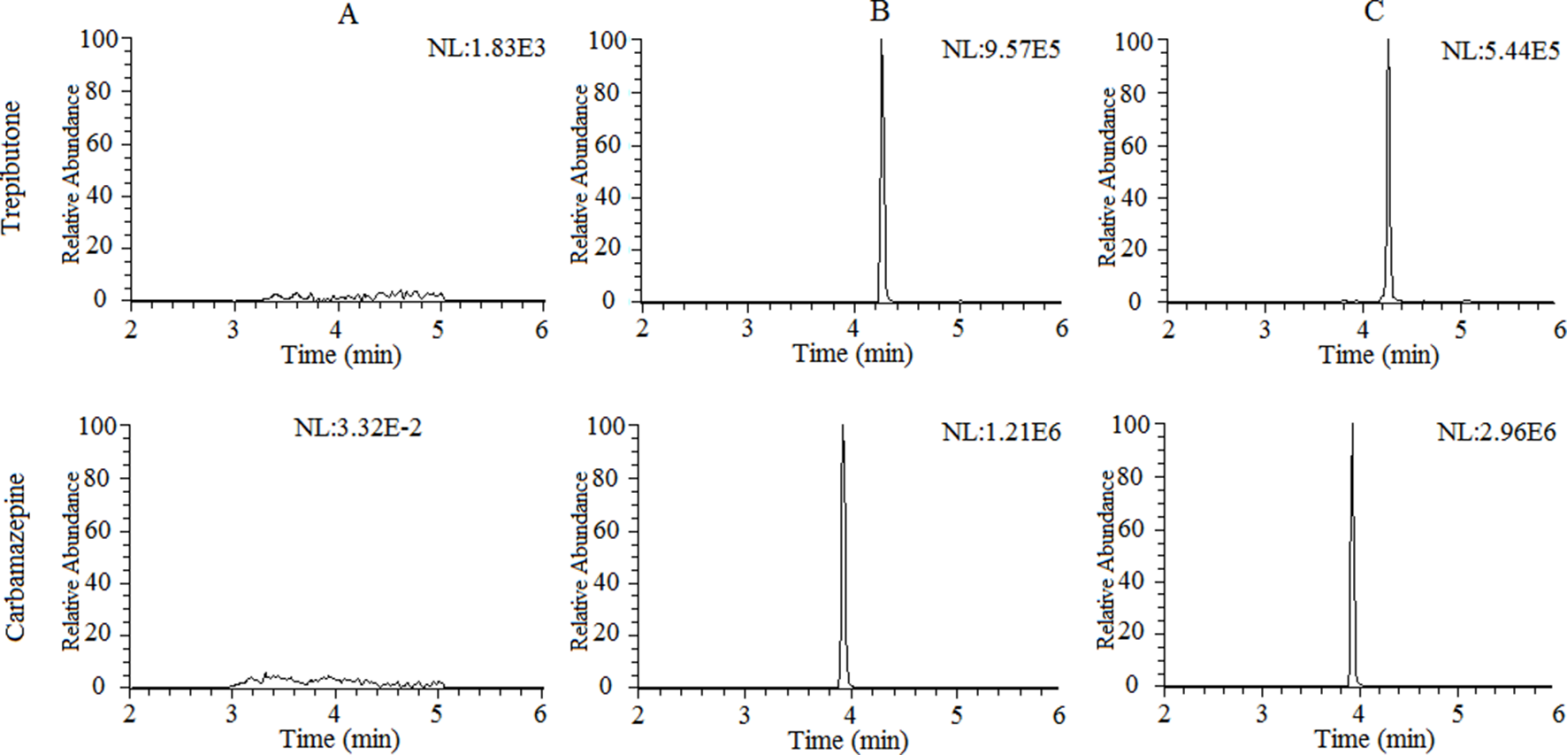
Representative chromatograms of trepibutone and IS in rat plasma: **(A)** Blank plasma,** (B)** blank plasma spiked with trepibutone and IS, and** (C)** plasma samples with oral administration of trepibutone.

The selectivity of the method towards endogenous plasma matrix was evaluated with the plasma of six rats. The typical chromatograms obtained from blank plasma, blank plasma spiked with the analyte and IS, and a plasma sample after oral administration of trepibutone are presented in **Figure 1**. Due to the efficient sample treatment and high selectivity of MRM, no endogenous interference from the plasma was observed under the described chromatographic conditions.

#### Linearity and Calibration Curve

The LLOQ was observed at 1 ng·ml^-1^ for trepibutone and the calibration curve was *Y = *4.90×10^-4^ *X *+ 1.048×10^-3^, *r* = 0.9970, which evaluated in the range of 1–1,000 ng·ml^-1^ (Y was the peak area ratio, X represented the relative concentrations, and r was the correlation coefficient). The result suggested that the correlation between the ratio of peak area and concentration was satisfied within the test ranges. The above information was summarized in **Table 1**.

**Table 1:** The regression equations, linear ranges and LLOQs for the determination of trepibutone in rat plasma.

**Analytes**	*Y* = **a***X***+b**	**r**	**Linear range (ng/ml)**	**LLOQ (ng/ml)**
Trepibutone	*Y* = 4.90×10^-4^*X* + 1.048×10^-3^	0.9970	1.0-1000	1.0

#### Precision and Accuracy

The QC samples at low, medium, and high concentrations and IS were used to assay intra- and inter-day precision and accuracy. The results are exhibited in **Table 2**. The intra- and inter-day precision (RSD%) of trepibutone were within the range of 2.68%−9.91% with the accuracy (RE%) between −2.04% and 7.87% for three QC levels of the analyte. The values of precision and accuracy were within the acceptable range.

**Table 2:** Precision and accuracy for trepibutone and IS in rat plasma (*n* = 18, 6 replicates per day for 3 days).

Analytes	Nominal concentration (ng/ml)	Intra-day			Inter-day		
		Measured concentration (ng/ml)	Precision (% RSD)	Accuracy (% RE)	Measured concentration (ng/ml)	Precision (% RSD)	Accuracy (% RE)
	1.0	1.02 ± 0.07	7.29	1.78	1.05 ± 0.10	9.91	5.42
Trepibutone	3.0	3.04 ± 0.19	6.28	1.35	3.09 ± 0.19	3.04	6.02
	400.0	398.83 ± 17.28	4.33	-0.29	391.84± 18.46	4.71	-2.04
	800	855.99 ± 24.23	2.83	7.00	862.97± 23.15	2.68	7.87

#### Extraction Recovery and Matrix Effect

The results of extraction recovery and matrix effect of trepibutone and IS are described in **Table 3**. For trepibutone in rat plasma, the mean absolute recoveries were 84.39%–88.48% (RSD: 3.61%-9.86%) at three QC levels. As for IS, the mean absolute recovery was 87.23%, suggesting the pretreatment method was reasonable and stable. The matrix effect was ranged from 96.76% to 102.40% (RSD: 3.77%-6.38%) for trepibutone at three QC levels in rat plasma, and was 96.76% (RSD: 3.77%) for IS. The results demonstrated that no significant matrix effect for analyte and IS was observed, which indicated that the ionization competition between the analyte and the endogenous co-elution was negligible.

**Table 3:** Matrix effect and extraction recovery for trepibutone and IS in rat plasma (*n* = 6).

Analytes	Spiked concentration (ng/ml)	Extraction Recovery		Matrix Effect	
		Mean (%)	RSD (%)	Mean (%)	RSD (%)
Trepibutone	3.0	88.48	9.86	98.23	5.81
	400.0	87.19	3.61	98.29	6.38
	800	84.39	7.08	102.40	5.19
carbamazepine	50	87.23	7.11	96.76	3.77

#### Stability

The stability was studied in different conditions such as 6 h exposure at room temperature, three freeze/thaws cycles, 24 h storage at 4 °C, and 14 days storage at −20 °C. The whole process was conducted through six replicates of plasma samples at three QC levels. The post-preparation stability was studied through the extracted QC samples kept in the auto-sampler at 10 °C for 24 h. The results are shown in **Table 4**, and the accuracy (RE%) and precision (RSD%) were within the acceptable range for all within ±15%.

**Table 4:** Stability of trepibutone in rat plasma under different storage conditions (*n* = 6).

Analytes	Spiked concentration (ng/ml)	Room temperature		Auto-sampler 24h		Three freeze-thraw cycles		Stored at 4°C 24h		Storage at -20 °C 14 days	
		RE (%)	RSD (%)	RE (%)	RSD (%)	RE (%)	RSD (%)	RE (%)	RSD (%)	RE (%)	RSD (%)
	3.0	6.65	9.06	6.21	7.91	5.44	8.30	3.74	2.95	2.29	5.81
Trepibutone	400.0	-3.25	2.86	-3.39	3.08	-3.22	3.38	-0.85	3.12	-1.89	4.30
	800	7.84	2.66	8.52	4.16	7.49	2.98	6.74	2.55	9.11	3.64

#### Dilution Test

The precision value of the 30-dilution folds sample for trepibutone was 1.97% with the accuracy values (RE) -2.23%. The result showed that the study samples whose concentrations were above ULOQ could be diluted adequately with blank plasma and re-analyzed using any of the tested dilution factors

### Pharmacokinetic Study

**Figure 2:**
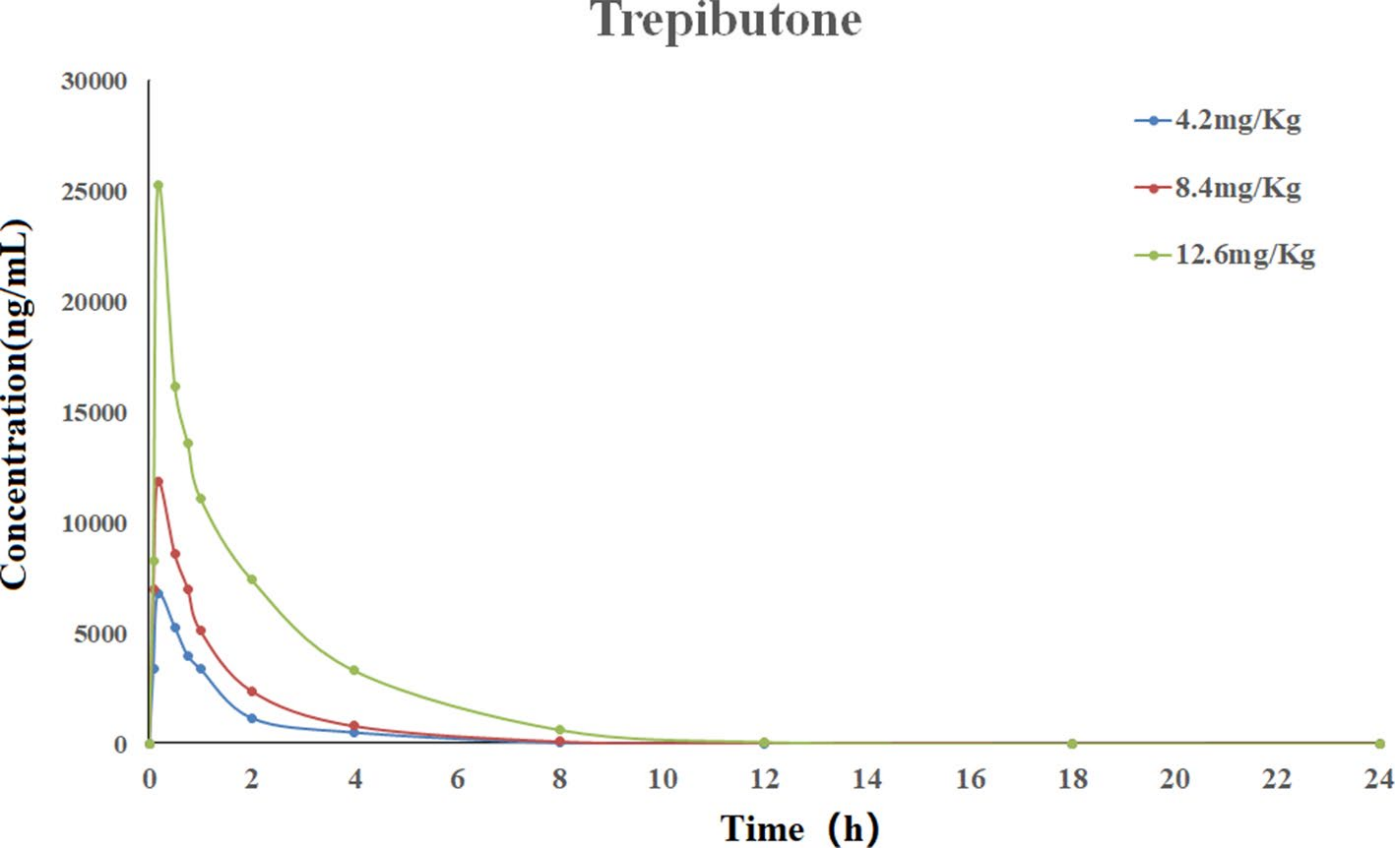
Mean plasma concentration-time profile of trepibutone after oral administrations of 4.2, 8.4, 12.6 mg·kg^-1^.

The validated UHPLC–MS/MS method was applied for the pharmacokinetic study of trepibutone. The rats took trepibutone orally at three doses of 4.2, 8.4, and 12.6 mg·kg^-1^. The data processing of pharmacokinetic parameters was in virtue of DAS 2.0 software in each administration group. The statistical analysis was conducted through the SPSS software. The drug concentration–time curve of trepibutone in plasma is shown in **Figure 2**, and the main pharmacokinetic parameters are summarized in **Table 5**. As seen from **Table 5**, the plasma concentrations of trepibutone increased rapidly after the oral administration of three doses and reached the *C* *_max_* at about 0.17 h, then the concentrations decreased, indicating that trepibutone was absorbed quickly. The *t* *_1/2_* values of trepibutone were within 4.73 ± 2.65 h, which suggested that trepibutone could be eliminated rapidly from the body, and there was no significant difference among three dose groups. Besides this, the linear regression analysis on the oral administration data showed that *C* *_max_*, *AUC* *_0-t_*, and *AUC* *_0-∞_* were dosage-dependent within the doges from 4.2 to 12.6 mg·kg^-1^.

**Table 5:** Pharmacokinetic parameters of Trepibutone after oral administrations at a dosage of 4.2, 8.4, 12.6 mg/kg.

Dosages (mg/kg)	Parameters				
	C_max_ (灜µg/ml)	*T*_max_ (h)	*T*_1/2_ (h)	AUC_(0-_ *_t_*_)_ (µg h/ml)	AUC_(0-∞)_ (灜µg h/ml)
4.2	6.77 ± 2.58	0.17	4.28 ± 1.33	10.27 ± 1.26	10.27 ± 1.26
8.4	11.86 ± 3.79	0.17	4.75 ± 1.09	17.08 ± 2.74	17.09 ± 2.74
12.6	25.26 ± 2.70	0.17	4.73 ± 2.65	45.33 ± 7.20	45.34 ± 7.19

### Metabolite Identification

#### Mass Spectrometric Behavior of Trepibutone

**Figure 3:**
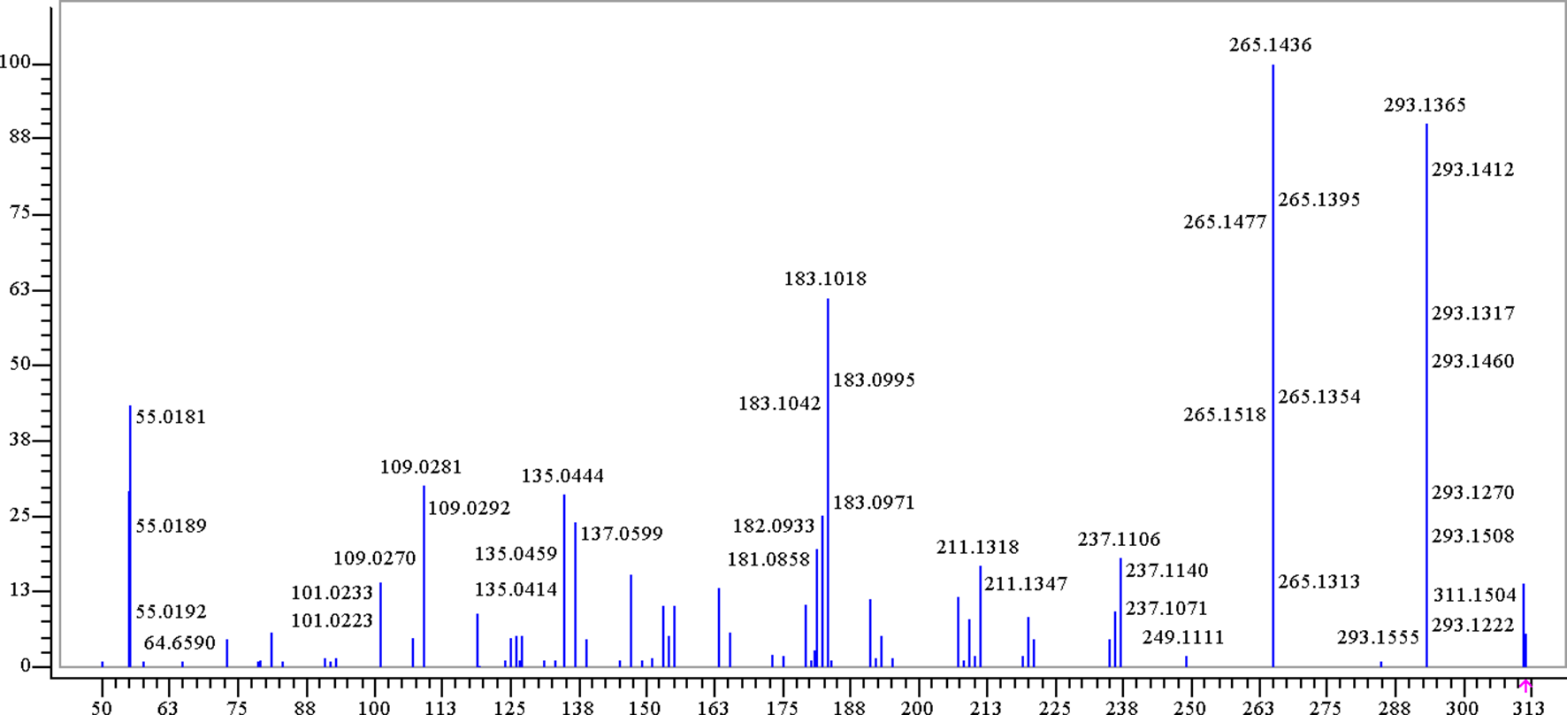
The product spectrum of trepibutone in positive electrospray ionization mode.

**Figure 4:**
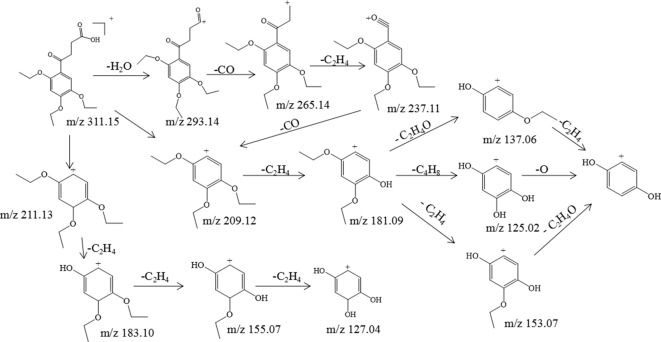
The proposed fragmentation pathway of trepibutone.

Trepibutone (C_16_H_22_O_6_) is a 4-oxo-4-(2,4,5-triethoxyphenyl) butanoic acid, which was detected at 27.728 min with *m/z* 311.14859, and it tended to lose H_2_O to generate the fragment ion C_16_H_21_O_5_ (*m/z* 293.14) due to the carboxyl group, and the crack happened with the loss of CO_2_ to produce the ion C_15_H_21_O_4_, of which the mass charge ratio was 265.14. The further loss of the alkyl group side chain was followed by the triethoxybenzene leaving. Next, the ethyl groups could break away during the process of the collision with inert gas in the collision cell. There were also some unexpected fragment behaviors appearing in the MS/MS spectrogram (**Figure 3**) of the trepibutone. For instance, the ion with *m/z* 211.13 possessed the structure of the triethoxy cyclohexadiene, and then the ethyl groups left gradually to generate fragment ions *m/z* 183.10, 155.07, and 127.04. The major fragments are shown as **Figure 4**.

#### Annotation of the Metabolites

The metabolite-related data was obtained from the UHPLC-HRMS and processed by the CD. The processed data contained formula, molecular weight, and potential transformations, which contributed to the prediction of the metabolite structures and the further understanding of the biotransformation. All metabolite information of trepibutone is shown in **Table 6**. And the extraction flow chromatographys of trepibutone and metabolites are listed in **Figure S1**.

**Table 6:** Metabolite information of trepibutone detected in plasma and annotated *via* UHPLC-Q-Orbitrap HRMS and data-mining tools in tandem.

No.	Formula	TR (min)	Peak Area	ES/expected (m/z)	ES/measured (m/z)	Delta (ppm)	Assignment	Fragment Ion(m/z)
parent	C_16_ H_22_ O_6_	27.728	9576113383	311.14891	311.14859	-1.044		293.14,265.14,237.11,211.13, 209.12,183.10,181.09,155.07, 153.05,137.06,127.04125.02, 109.03
M1	C_14_ H_16_ O_5_	25.414	9195753	265.10705	265.10724	0.716	-(C_2_ H_6_ O)	237.11,209.08,191.07,181.09,163.04,153.05,147.04,135.04,119.05,107.05
M2a	C_14_ H_18_ O_6_	25.414	176729214	283.11761	283.11795	1.184	-(C_2_ H_4_)	265.11,237.11,209.08,191.07, 183.10,163.04,155.07,147.04,137.06,135.04,119.05,109.03,107.05
M2b	C_14_ H_18_ O_6_	27.064	140325458	283.11761	265.10681	-0.906	-(C_2_ H_4_)	265.11,237.08,209.08,191.07,183.10,181.05,163.04,155.07,153.02,135.04,127.04,109.03
M3	C_16_ H_20_ O_5_	27.735	233480804	293.13835	293.13821	-0.478	-(H_2_ O)	265.14,249.11,237.11,207.07,191.07,181.09,163.04,153.05,147.04,135.04,107.05
M4	C_15_ H_20_ O_6_	26.763	6088472	297.13326	297.13202	-4.189	-(C H_2_)	279.12,251.13,223.10,197.11,177.05,169.09,149.06,123.04
M5	C_16_ H_20_ O_6_	28.293	210462489	309.13326	309.13297	-0.954	-(H_2_)	291.12,263.13,248.10,235.10,220.11,209.12,192.08,180.07,163.04,152.05,135.04,109.03
M6	C_17_ H_24_ O_6_	29.659	185461344	325.16456	325.16336	-3.706	+(C H_2_)	293.14,265.14,237.11,207.07,181.09,163.04,115.04
M7	C_16_ H_22_ O_7_	25.205	350551505	327.14382	327.14352	-0.946	+(O)	309.13,281.14,252.10,236.09, 227.13,207.07,199.10,191.07,179.03,163.04,153.05,135.04,109.03
M8	C_16_ H_20_ O_8_	25.426	5224112	341.12309	341.12259	-1.478	-(H_2_) +(O_2_)	323.11,295.11,267.09,241.11,221.08,207.07,195.10,180.08,166.06,151.04,135.04,111.04

**Figure 5:**
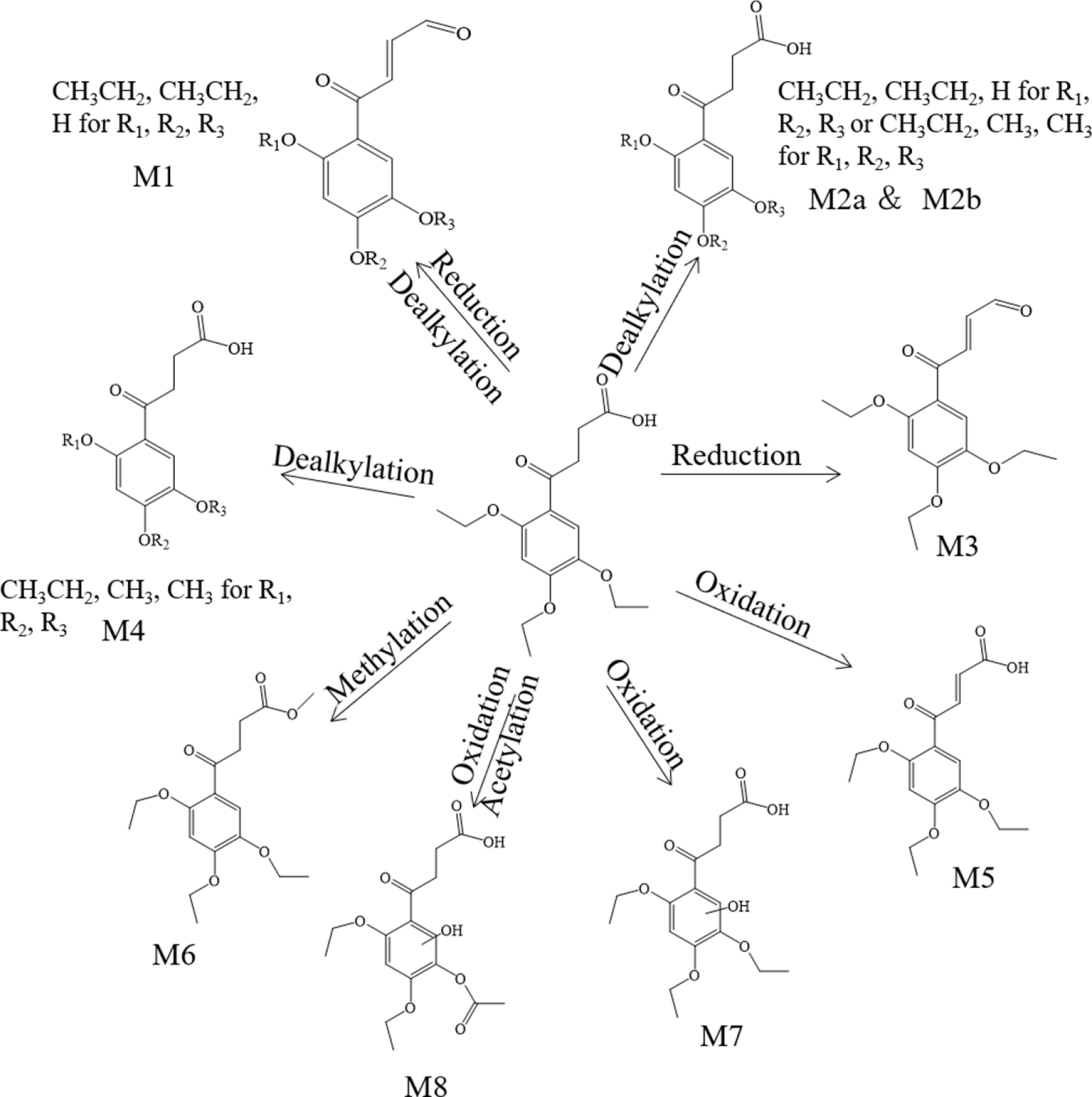
The proposed metabolites of trepibutone in the plasma.

**Metabolite M** **_1_** **.** The molecular formula detected for the metabolite M_1_ was C_14_H_16_O_5_ with measured *m/z* 265.10724. Compared with the trepibutone (C_16_H_22_O_6_), only a difference of C_2_H_6_O was observed. Due to the carboxyl group existing in the parent drug, it was reasoned that the loss of H_2_O was conducted in the collision pool along with the alkyl group leaving from the triethoxy group. The conjectural structure for the metabolite M_1_ is shown in **Figure 5**. And there was a deviation of 28 Da between the ions at *m/z* 237.11, 209.08 and 181.09, which suggested two ethyl groups. Besides, the neutral loss of H_2_O was observed when the oxygen broke away, and the previous expectation was validated by comparing it with spectra.

**Metabolite M** **_2_** **.** The molecular formula for the metabolite M_2_ (C_14_H_18_O_6_) indicated a dealkylated step from the trepibutone (C_16_H_22_O_6_). Based on the structure characteristics, the dealkylation of the triethoxy was obvious. The M_2_ was found at 25.414 min with *m/z* 283.11795 and at 27.064 min, suggesting that there were two isomers (M_2a_ and M_2b_) for the C_14_H_18_O_6_. The spectra of both gave the fragment ions at *m/z* 265.14, 237.11, 183.10, 163.04, 137.06, 109.03, 101.02, 73.03, and 55.02, which were in accordance with the parent drug, illustrating that the nuclear structure remained the same.

The difference between the metabolite M_2_ and the trepibutone was a C_2_H_4_; in addition to the core structure, the other part exhibited disparity based on the spectrogram. Therefore, the structures for the M_2a_ and M_2b_ were speculated as one that removed two methyl groups and one that removed diethyl products. The structures for M_2_ can be seen in **Figure 5**.

**Metabolite M**_3_ **. **The metabolite M_3_ was displayed in the positive mode with a molecular formula of C_16_H_20_O_5_. This metabolite originated from the dehydration procedure with the neutral loss of H_2_O. Due to the first step of losing H_2_O from the trepibutone in the product ion spectrum, it was assumed that the spectrum behavior of the metabolite was in accordance with the parent drug, and, in fact, that was the case. Hence, this metabolite was identified as the dehydration product of the carboxyl group.

**Metabolite M**_4_ **.** The formula calculated* via *accurate molecular weight for this metabolite was C_15_H_20_O_6,_ which was eluted at 26.763 min with *m/z* 297.13202. From the view of the structural formula, the metabolite M_4_ was a demethylation product and the product ion spectrum of this structure was validated through the analysis tool. Then, this metabolite was identified provisionally and it is shown in **Figure 5**.

**Metabolite M**_5_ **.** The molecular formula for the M_5_ (C_16_H_20_O_6_) suggested that just a reduction process was carried out to obtain this metabolite. The M_5_ was found at the 28.293 min with the *m/z* 309.13297, and the fragment ions (*m/z* 209.12) existed in the spectrum of the M_5_, indicating that the triethoxybenzene ring remained unchanged, and that the ions (*m/z* 291.12 and 263.13) were two Da less than product ions (*m/z* 293.14 and 265.14) in the trepibutone suggested that the hydrogen atoms left from the C_2_ and C_3_ of the butanoic acid. This reduced product was proposed as the feasible structure by analyzing fragmentation information.

**Metabolite M**_6_ **.** The M_6_ showed a molecular formula of (C_17_H_24_O_6_), which implied a phase II metabolic reaction (methylation) for only an increase of CH_2_ on the base of trepibutone. This metabolite was detected at 29.659 min with *m/z* 325.16336. The fragment ion (*m/z* 293.14) was 32 Da less than the parent ion, which illustrates the existence of the methoxy group, and the carboxyl group was the most suitable location to conduct the methylation. Through the analysis and validation of the software the result accorded with the hypothesis.

**Metabolite M**_7_ **.** The *m/z* of M_7_ was 327.14352 with the molecular formula C_16_H_22_O_7_, which implied an oxidation relative to the trepibutone (C_16_H_22_O_6_). There were several potential oxidation sites, among which the oxidation in the benzene ring was more reasonable. The behavioral tendency to lose water and an ethyl group was coincident with the trepibutone. And the structure of this metabolite was exported to the MF to crack under simulated condition based on the database. The result revealed that the structure we analyzed matched the fragment ion spectrum to a great extent.

**Metabolite M**_8_ **.** This metabolite was detected at 25.426 min with *m/z* 341.12259, just 14 Da more than the M_7_ (C_16_H_22_O_7_), and the molecular formula calculated was C_16_H_20_O_8_. Thus, we conjectured an increase of carbonyl oxygen on the basis of the M_7_. And the acetylation was the principal reaction type during the process of metabolism, and, consequently, it was reasoned that the M8 had undergone acetylation with the loss of an ethyl group. Through the comparison of structural characteristics and fragment information, we determined that this was the most feasible metabolic pathway to obtain M_8_.

**Figure 6:**
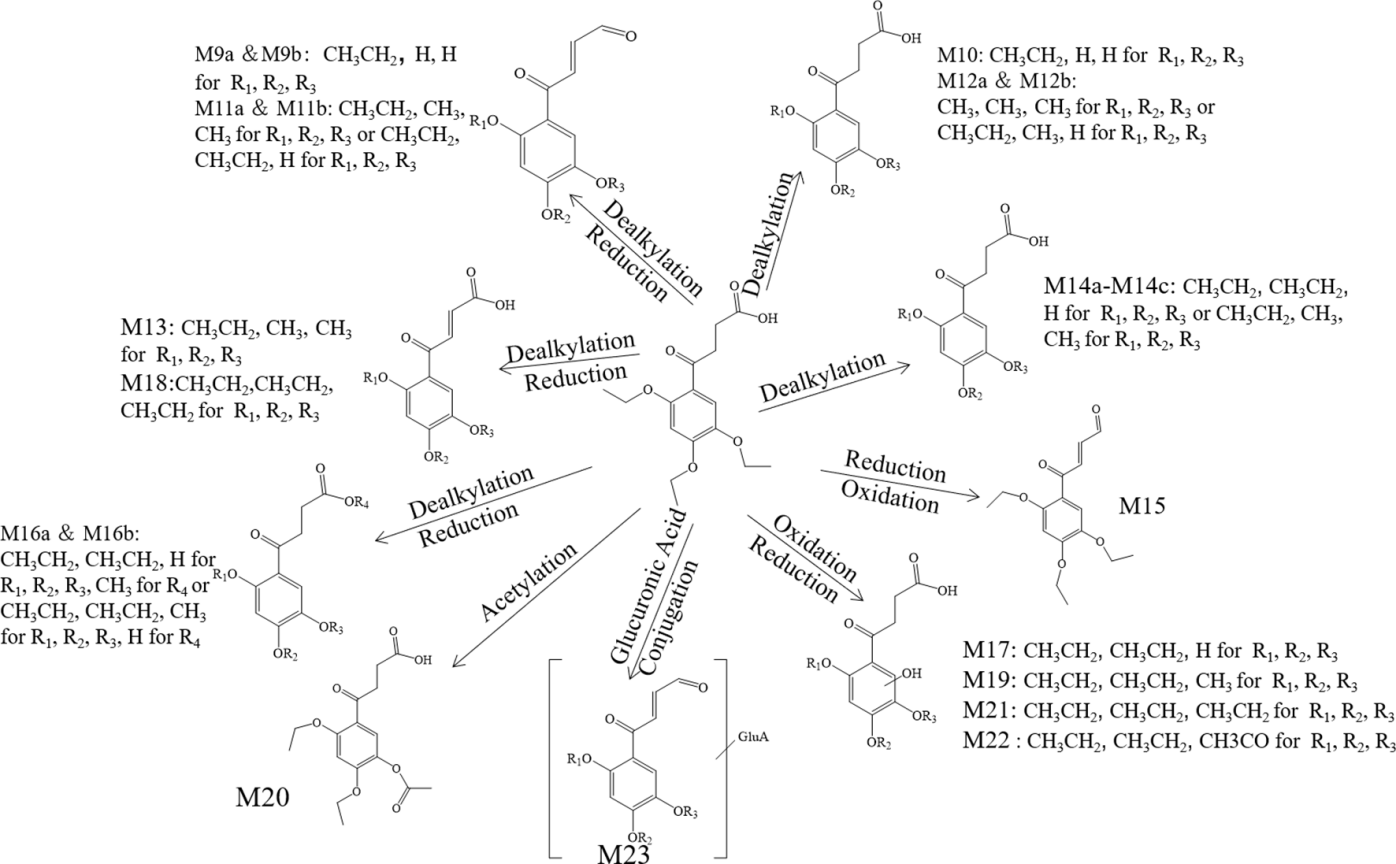
The proposed metabolites of trepibutone in the urine.

**Metabolite M**_9_ **.** The metabolite M_9_ was detected in the positive mode, and the molecular formula fitting for the M_9_ (C_12_H_12_O_5_) suggested the loss of the H_2_O compared to the trepibutone (C_16_H_22_O_6_), which possessed a carboxyl group. Then, the difference of C_4_H_10_ might show that two ethyl groups left as the reduction happened simultaneously. However, we detected the C_12_H_12_O_5_ at 23.946 and 25.408 min separately, which indicated there were two isomers (M_9a_ and M_9b_) for C_12_H_12_O_5_. The structure inferred was coincided with the mass spectrometric behavior and is shown in **Figure 6**.

**Metabolite M**_10_ **.** The molecular formula acquired for the M_10_ (C_12_H_14_O_6_) indicated the loss of two ethyl groups from the triethoxyphenyl group of the trepibutone. Metabolite M_10_ was detected at 24.209 min with *m/z* 255.08716. Compared with the spectrogram of M_9_, most of the fragment ions were common after the loss of H_2_O in metabolite M_10_. Therefore, it could be speculated that M_9_ originated from the M_10_ through dehydration.

**Metabolite M**_11_ **.** Metabolite M_11_ exhibited a molecular formula of C_14_H_16_O_5_ and was found at 25.406 and 27.057 min, similarly to the M_9_ and M_10._ Metabolite M_11_ had two isomers (M_11a_ and M_11b_). Besides, M_11_ had just one C_2_H_4_ more than M_9_, so we conjectured that M_11_ possessed the analogous cracking behavior with the M_9,_ and the results agreed with this. The potential metabolites M_11a_ and M_11b_ are shown in **Figure 6**.

**Metabolite M**_12_ **.** The M_12_ displayed a formula (C_13_H_16_O_6_) with a distinction of C_3_H_6_ between this metabolite and trepibutone (C_16_H_22_O_6_), which indicated the dealkylation: the representative phase I metabolism. According to the structural characteristics, the loss of an alkyl group could only be carried out at the site of an oxyethyl group in the benzene ring. 

The data acquired from the high sensitivity mass spectrometry showed that the C_13_H_16_O_6_ was detected at 24.229 and 21.491 min, illustrating that the M_12_ might possess two structures (M_12a_ and M_12b_). Through comprehensive analysis of the above information, it was assumed that there were two situations in theory, one was the loss of CH_3_ from three ethoxy groups separately, the other was the leaving of C_2_H_4_ and CH_3_ from two arbitrary ethoxy groups.

**Metabolite M**_13_ **.** Metabolite M_13_ exhibited the molecular formula C_14_H_16_O_6_ found at 25.769 min, and a difference of C_2_H_6_ was observed compared with the trepibutone. Considering the structure of trepibutone, we conjectured that demethylation happened *in vivo* to generate the M_13_. And the potential metabolites M_13_ are shown in **Figure 6**.

**Metabolite M**_14_ **.** The data acquired showed that M_14_ was detected at 27.057, 24.442, and 25.465 min, illustrating that the M_14_ might possess three structures (M_14a_, M_14b_ and M_14c_), and the fitted molecular formula was C_14_H_18_O_6_, which is just one difference between C_2_H_4_ and trepibutone. This metabolite was detected in the plasma (metabolite M_2_) at the 25.414 and 27.064 min as well.

The MS^2^ spectrogram showed that the above metabolites possessed a mutual core structure. As described in the metabolite M_2_, these metabolites were the products of demethylation or deethylation. Due to three ethoxy groups in the trepibutone, there were three possible structures for demethylation and three different structures for deethylation, among which three isomers were detected by the high resolution mass spectrometry.

**Metabolite M**_15_ **.** The molecular formula obtained for the M15 (C_16_H_20_O_5_) also suggested the process to lose the water had a difference of 18 Da compared with the trepibutone, and the only possible position in which to lose H_2_O was the carboxyl group. This metabolite was detected at the 27.728 min with *m/z* 293.13818, and the retention time was close to that of the metabolite M_3_, which also was the product of the dehydration. By comparing the spectrums, it seemed that the M_15_ was the same substance as the metabolite M_3_.

**Metabolite M**_16_ **.** The predicted molecular formula for the metabolite M16 was C_15_H_20_O_6_. This metabolite was detected at 26.748 and 27.083 min separately, meaning that there were two isomers (M_16a_ and M_16b_).

In the plasma, C_15_H_20_O_6_ was also detected at 26.763 min (metabolite M_4_), and the mass spectrogram was highly analogous with M_16a_, and we thus belived M_4_ and M_16a_ were the same substance. For metabolite M_16b_, the analytical tool suggested the process of methylation and dealkylation. Through comprehensive manifold information, the structure of M_16b_ is shown as **Figure 6**.

**Metabolite M**_17_ **.** The fitted molecular formula for M_17_ was C_14_H_18_O_7_, which was detected at 25.868 min with mass charge ratio 299.11252. The analytical technique suggested that this metabolite underwent the process of dealkylation and oxidation, we conjectured that an ethyl group was taken off and oxidation happened in the benzene ring based on the structural feature. We assumed the structure matched with its MS^2^ spectrum, and this was confirmed* via *MF. Thus, the structure of metabolite M_17 _was identified it is shown in **Figure 6**.

**Metabolite M**_18_ **.** The M_18_ showed a molecular formula of C_16_H_20_O_6_, losing two hydrogen atoms compared with the trepibutone (C_16_H_22_O_6_) which implied a dehydrogenation in the process of metabolism. And that was more appropriate if the reduction was carried out by the butyric acid. In addition to this, such a metabolite seemed to be found in the plasma as well, and through comparing the spectrum, retention time, and mass charge ratios of this metabolite in the plasma and urine, the M_18_ and M_5_ were found to be the same material.

**Metabolite M**_19_ **.** The M_19_ with the molecular formula C_15_H_20_O_7 _was detected at 24.138 min with *m/z* 313.12720. From the view of molecular composition, M_19_ had an increase in oxygen atoms and a decrease in carbon atoms. Based on the metabolic regularity *in vivo*, we assumed that demethylation and oxidation occurred to generate this metabolite. This hypothesis was supported by the product ion spectrum.

**Metabolite M**_20_ **.** The M_20_ was detected at the 28.831 min with *m/z* 325.12756. This metabolite showed a molecular formula of (C_16_H_20_O_7_); only an increase of 14 Da on the base of trepibutone, this implied a phase II metabolic reaction–acetylation after the loss of an ethyl group. The MS^2^ spectrum was in accordance with the speculative structure, which was confirmed by MF. Detailed information can be seen in **Table 7** and the structure in **Figure 6**.

**Table 7:** Metabolite information of trepibutone detected in urine and annotated *via* UHPLC-Q-Orbitrap HRMS and data-mining tools in tandem.

No.	Formula	TR (min)	Peak Area	ES/expected (m/z)	ES/measured (m/z)	Delta (ppm)	Assignment	Fragment Ion(m/z)
parent	C_16_ H_22_ O_6_	27.728	1693832709	311.14891	311.14859	-1.044		293.14,265.14,237.11,211.13,209.12,183.10,181.09,163.04,155.07,153.05,137.06,127.04,125.02,109.03
M9a	C_12_ H_12_ O_5_	23.946	66692573	237.07575	237.07562	-0.548	-(C_4_ H_10_ O)	209.08,191.07,181.05,163.04,153.02,135.04,111.04
M9b	C_12_ H_12 _O_5_	25.408	17612099	237.07575	237.07666	3.838	-(C_4_ H_10_ O)	209.08,207.07,191.07,179.03,163.04,153.05,147.04,135.04,119.05,107.05
M10	C_12_ H_14_ O_6_	23.92	301845145	255.08631	255.08716	3.314	-(C_4_ H_8_)	237.08,209.04,181.05,163.04,153.02,135.04,127.04,109.03
M11a	C_14 _H_16_ O_5_	27.057	103702859	265.10705	265.10675	-1.132	-(C_2_ H_6_ O)	237.08,209.08,191.07,181.05,173.02,163.04,153.02,135.04,111.04
M11b	C_14_ H_16_ O_5_	25.406	1619096554	265.10705	265.10678	-1.019	-(C_2_ H_6_ O)	237.11,221.08,209.08,191.07,181.09,163.04,153.05,147.04,135.04
M12a	C_13 _H_16_ O_6_	24.229	91995418	269.10196	269.10208	0.428	-(C_3_ H_6_)	251.09,223.10,195.07,169.09,163.08,154.06,147.04,141.05,135.04,126.03,119.05,109.03
M12b	C_13_ H_16_ O_6_	21.491	134046831	269.10196	269.10172	-0.909	-(C_3_ H_6_)	251.09,223.10,195.07,169.09,163.08,154.06,141.05,147.04,135.04,119.05,109.05,107.05
M13	C_14_ H_16_ O_6_	25.769	20388075	281.10196	281.10147	-1.76	-(C_2_ H_6_)	263.09,235.10,207.07,192.08,179.07,163.04,146.04,135.04,107.05
M14a	C_14_ H_18 _O_6_	27.03	1027617434	283.11761	283.11563	-7.01	-(C_2_ H_4_)	265.11,237.08,209.08,191.07,181.05,163.04,155.07,135.04,127.04, 109.04
M14b	C_14 _H_18 _O_6_	24.442	97925053	283.11761	283.11749	-0.441	-(C_2_ H_4_)	265.11,237.11,209.08,191.07,183.10,163.04,155.07,147.04,135.04,119.05,109.03
M14c	C_14_ H_18 _O_6_	25.465	28442168447	283.11761	283.11789	0.972	-(C_2_ H_4_)	265.11,237.11,209.08,191.07,183.10,163.04,155.07,147.04,135.04, 119.03
M15	C_16_ H_20_ O_5_	27.728	39690973	293.13835	293.13818	-0.581	-(H_2_ O)	265.14,237.11,220.11,207.07,191.07,181.09,163.04,153.05,147.04,107.05
M16a	C_15_ H_20 _O_6_	26.748	11654649	297.13326	297.13675	11.729	-(C H_2_)	279.12,251.13,223.10,197.12,169.08,147.04,141.05,135.04,123.04,109.03
M16b	C_15 _H_20_ O_6_	27.083	6893018	297.13326	297.13779	15.229	-(C H_2_)	237.11,209.08,183.10,153.02,135.04,109.03
M17	C_14_ H_18_ O_7_	25.868	34638435	299.11252	299.11252	-0.031	-(C_2_ H_4_) +(O)	281.10,253.11,225.08,209.08,199.10,179.03,171.07,163.04,153.06,143.03,125.02,109.03
M18	C_16_ H_20_ O_6_	25.16	716678901	309.13326	309.13287	-1.277	-(H_2_)	291.12,263.13,248.10,235.10,220.11,209.12,192.08,180.08,163.04,152.05,147.04,135.04,109.03
M19	C_15_ H_20_ O_7_	24.138	23479362	313.12817	313.1272	-3.128	-(C H_2_) +(O)	295.12,267.12,222.09,213.11,193.05,177.05,167.07,154.06,137.06,109.03
M20	C_16_ H_20_ O_7_	28.831	16915598	325.12817	325.12756	-1.905	-(H_2_) +(O)	279.12,251.13,237.11,223.06,209.08,195.03,181.05,153.02,135.04,109.03
M21	C_16 _H_22_ O_7_	25.189	36898098894	327.14382	327.14377	-0.182	+(O)	309.13,281.14,253.11,236.10,227.13,207.07,199.10,179.03,163.04,153.05,137.06,125.02,109.03
M22	C_16_ H_20 _O_8_	25.427	604232484	341.12309	341.12222	-2.562	-(H_2_) +(O_2_)	323.11,295.12,267.09,249.11,241.11,220.07,207.07,195.10,180.08,166.06,135.04,111.04
M23	C_20_ H_24_ O_11_	24.988	11901984	441.13913	441.13962	1.093	+(C_4 _H_2 _O_5_)	265.11,237.08,209.08,181.05,141.02,131.03

**Metabolite M**_21_ **.** The mass charge ratio of M_21_ was 327.14352 with the molecular formula C_16_H_22_O_7_, which implied an oxidation relative to the trepibutone (C_16_H_22_O_6_). It was found at 25.189 min. The retention time and fragment ions were similar to metabolite M_7_, and thus this metabolite and M_7_ might be the same substance. And the structure of this metabolite was exported to the MF to crack, the result revealed that the hypothesis was sound.

**Metabolite M**_22_ **.** This metabolite was detected at 25.427 min with *m/z* 341.12259, just 14 Da more than the M_21_ (C_16_H_22_O_7_), and the molecular formula calculated was C_16_H_20_O_8_. The retention time and precise molecular weight was analogous with M_8_, and thus we conjectured the structure of both was the same. This assumption was confirmed, and the structure can be seen in **Figure 6**.

**Metabolite M**_23_ **.** The M_23_ exhibited a molecular formula of C_20_H_24_O_11 _increasing by C_4_H_2_O_5_, and although there was no direct indication of glucuronide conjugate, the increasing amount of oxygen supported this point. And it was thought that deethylation and dehydration happened in order to produce this metabolite.

The M_23_ was acquired at 24.988 min with *m/z* 441.13962. The product ion (*m/z* 265.11) confirmed the presence of the glucuronide moiety by the typical loss of 176 Da in positive ionization mode. The ion of *m/z* 265.11 resembled the M_11_ *via *the analysis of fragments, and we therefore assumed the structure was similar to that of M_23_ as exhibited in **Figure 6**.

## Discussion and Conclusions

Trepibutone has been used in clinic for its excellent curative effect, but a lack of pharmacokinetic studies limits its clinical rational use. The established method in this article was accurate, sensitive, and repeatable, which was successfully applied to the study on the pharmacokinetics of trepibutone. These results indicated that the absorption was fast with an early T_max _of 0.2 h, and elimination was performed at a rapid speed with the T_1/2 _within 1.94–5.95 h. Meanwhile these pharmacokinetic parameters provided a theoretical basis for the reasonable use of trepibutone and individualized therapy.

This research provided a new method for the identification and characterization of metabolic reactions and analyzed whether the fragmentation patterns of metabolite structures was analogous to the parent drug. Therefore, the more clearly the fragmentation regulation of trepibutone was elucidated, the more favorable it was to identify metabolites. And as the development of analytic instrument, the detection of samples tended towards high sensitivity, high accuracy, and high-flux. The data obtained were multifarious, and thus it might be a future trend that artificial intelligence and big data banks would play an important role in metabolite identification.

In conclusion, an accurate and repeatable UHPLC-MS/MS method was developed for the analysis of trepibutone and the established method showed good performance in terms of linearity, LLOQ, precision, and accuracy, etc. This method was successfully applied to the pharmacokinetic study of trepibutone. The knowledge obtained could offer useful information for clinical application. The UHPLC-Q-Orbitrap HRMS and the multiple data-mining tools were used for metabolite exploration and identification. As a result, a total of 30 metabolites of trepibutone in plasma and urine were identified. Considering the low number of reports that exist on the metabolic profile of trepibutone, our paper will provide a theoretical basis for the further research.

## Data Availability Statement

All datasets generated for this study are included in the article/**Supplementary Material.**

## Ethics Statement

All protocols of animal experiments were in accordance with the Regulations of Experimental Animal Administration issued by the Animal Ethics Committee of the institution of First Affiliated Hospital of Zhengzhou University.

## Author Contributions

ZS, JY, JK, and LZuo conceived and designed the experiments. LL, YX, LZhou, QJ, YS, and XD carried out the experiments. JY, LL, and YX analyzed the data. ZS wrote the manuscript. JK and LZuo critically revised the manuscript. All the authors read and reviewed the final manuscript.

## Conflict of Interest

The authors declare that the research was conducted in the absence of any commercial or financial relationships that could be construed as a potential conflict of interest.

## Supplementary Material

Data Sheet 1 The extracted ion chromatograms of metabolites and trepibutone.
