# Supplementary figures and images for: Pharmacokinetics and Metabolite Profiling of Trepibutone in Rats Using Ultra-High Performance Liquid Chromatography Combined With Hybrid Quadrupole-Orbitrap and Triple Quadrupole Mass Spectrometers
Source: Front Pharmacol. 2019 Nov 4;10:1266. doi: 10.3389/fphar.2019.01266 (PMC6843799; doi:10.3389/fphar.2019.01266)

RT: 0.00 - 40.00

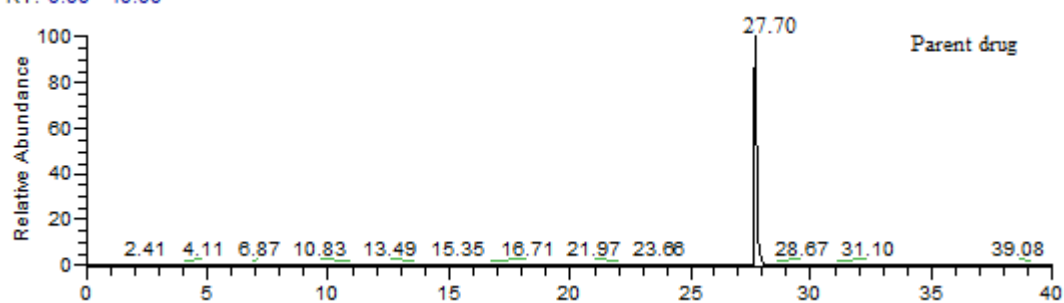

RT: 0.00 - 40.00

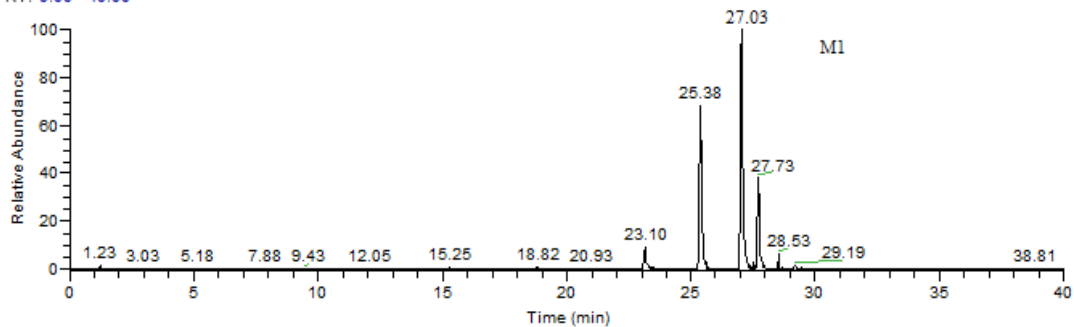

RT: 0.00 - 40.00

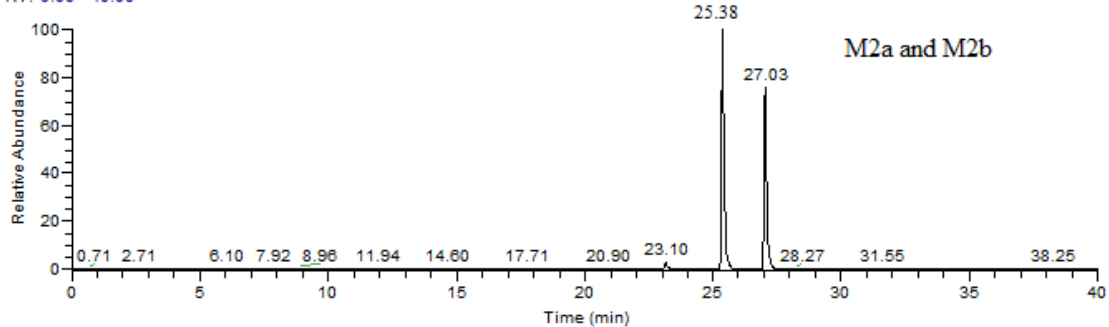

RT: 0.00 - 40.00

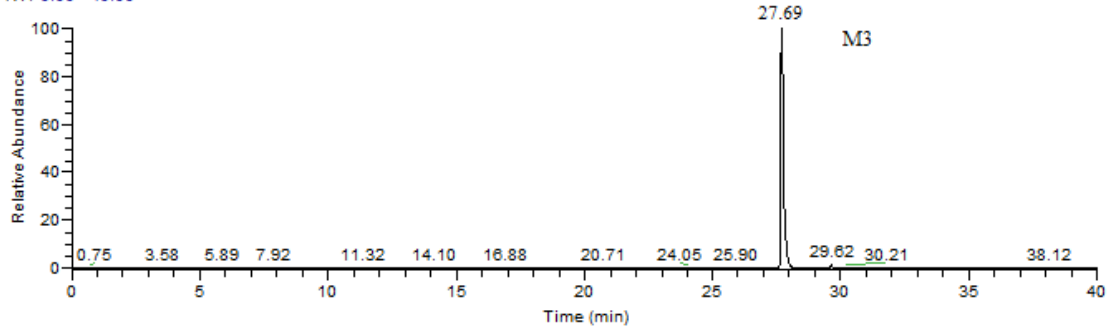

RT: 0.00 - 40.00

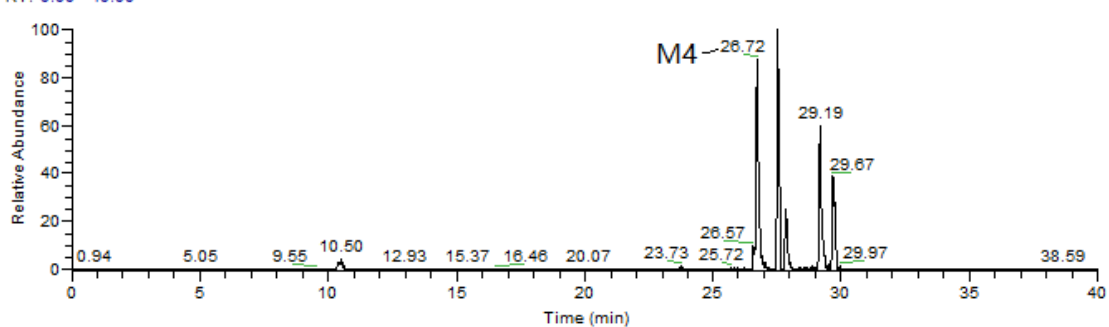

RT: 0.00 - 40.00

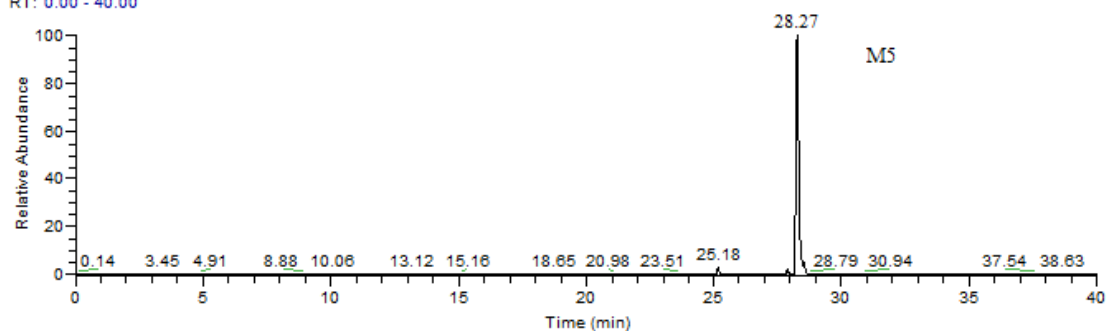

RT: 0.00 - 40.00

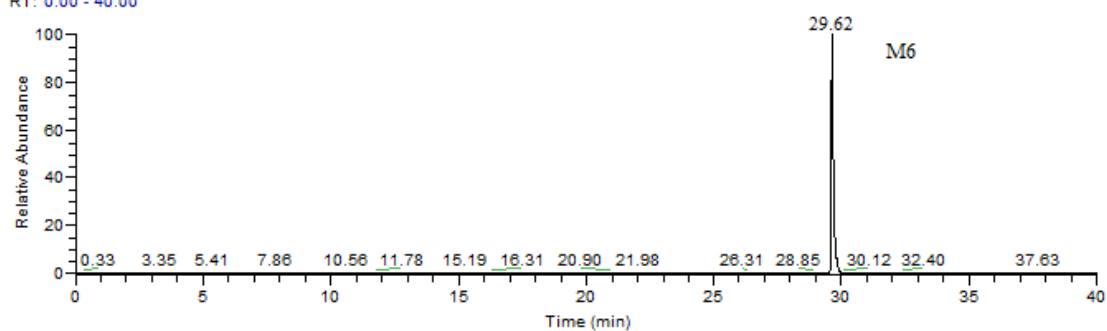

RT: 0.00 - 40.00

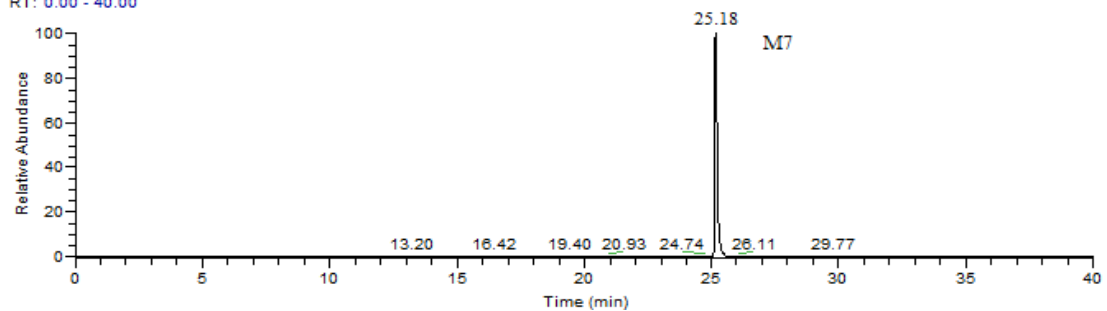

RT: 0.00 - 40.00

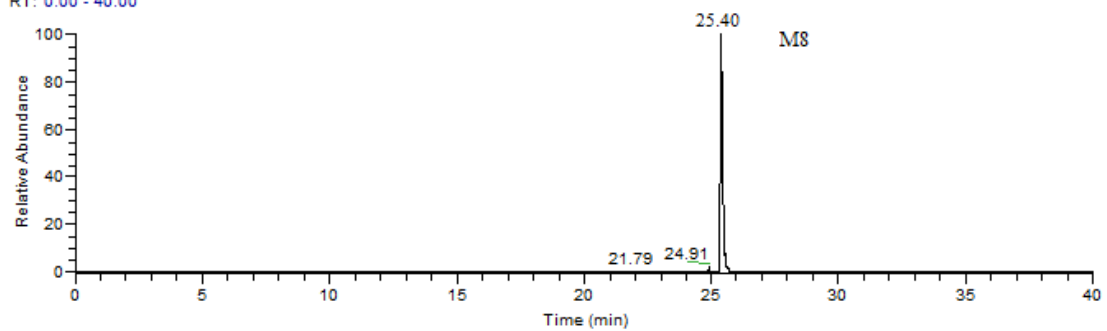

RT: 0.00 - 40.00

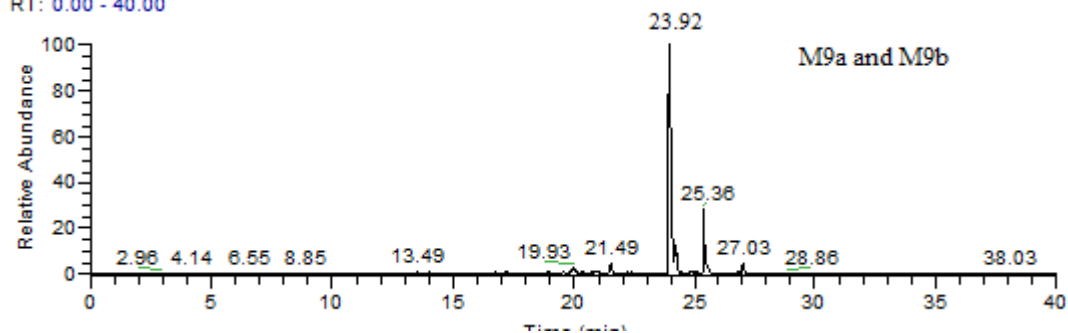

RT: 0.00 - 40.00

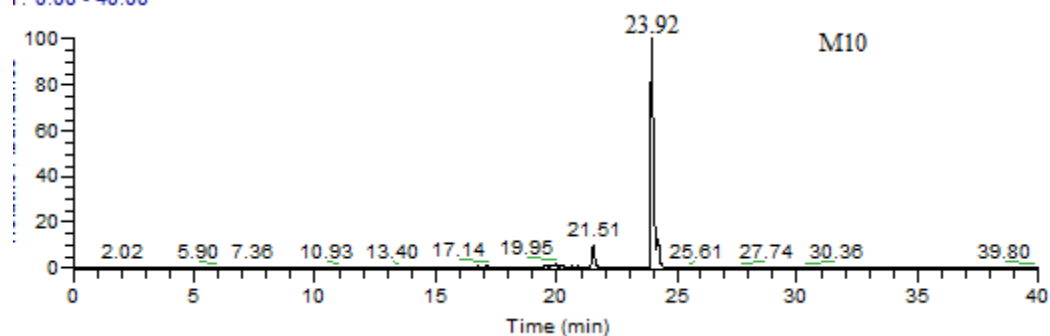

RT: 0.00 - 40.00

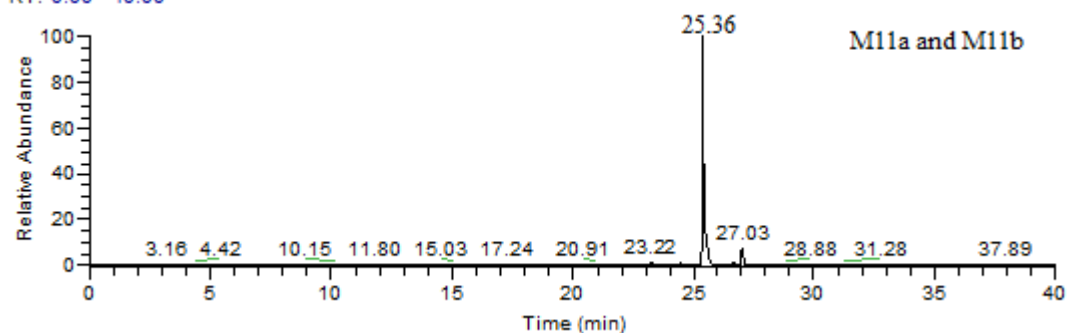

RT: 0.00 - 40.00

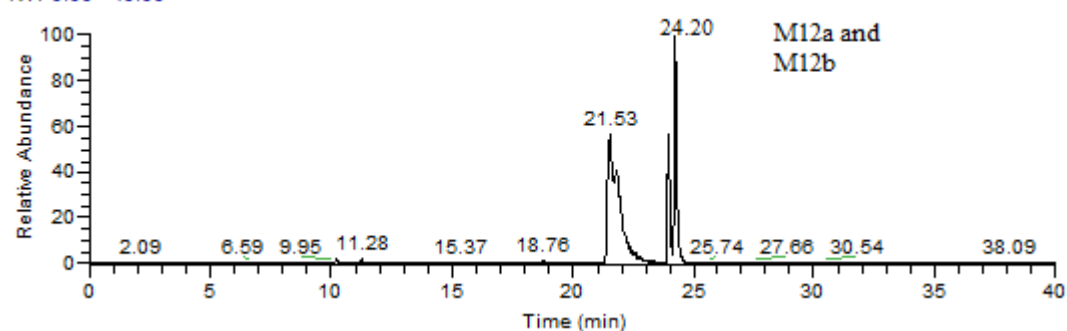

RT: 0.00 - 40.00

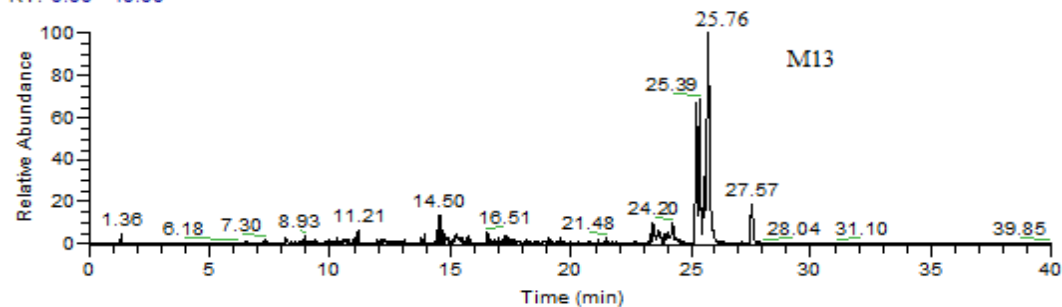

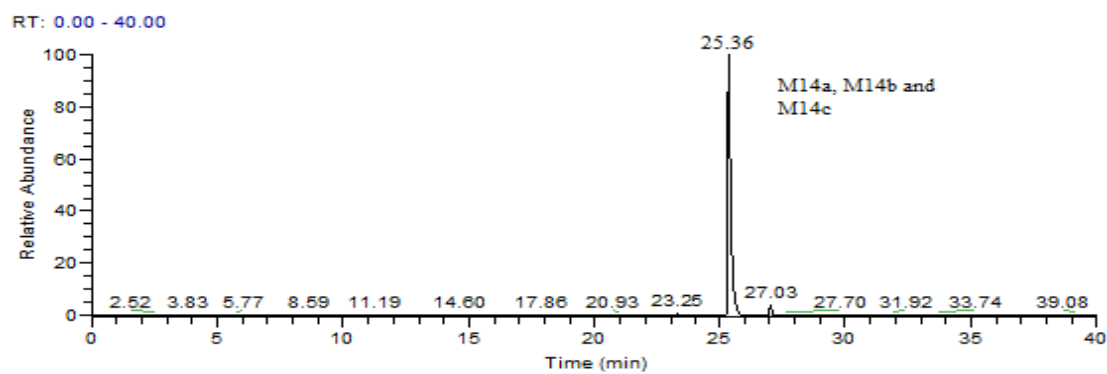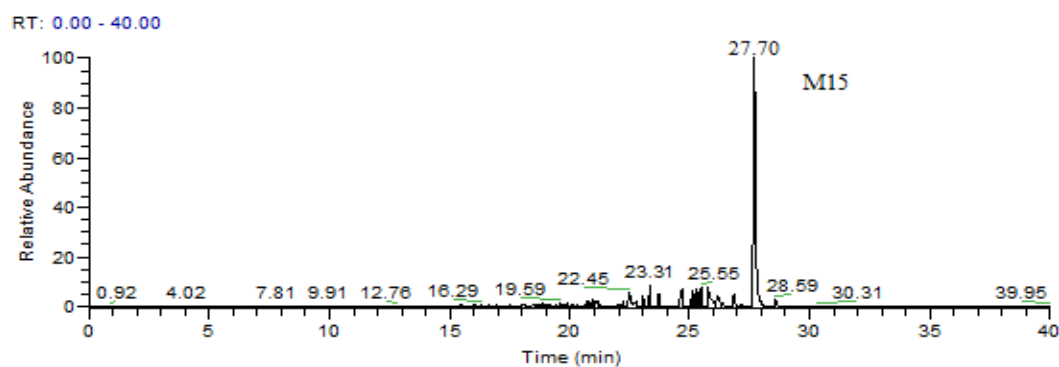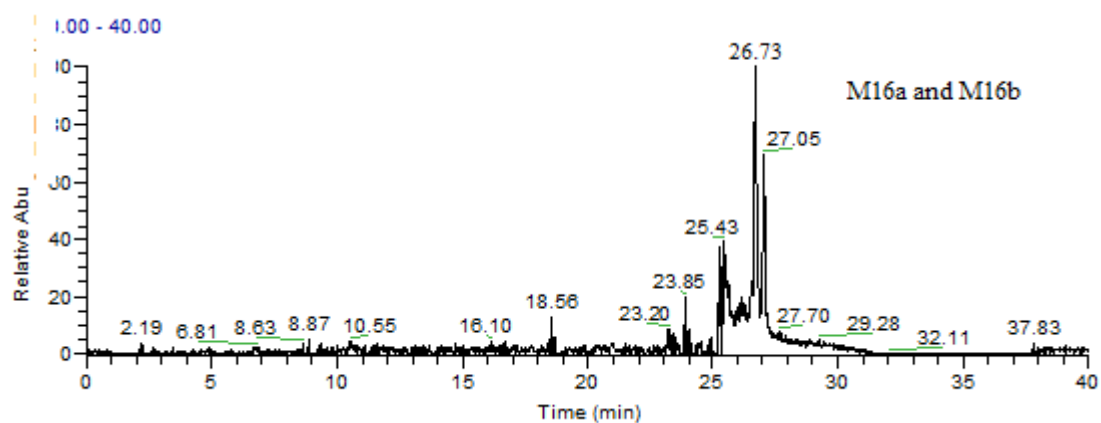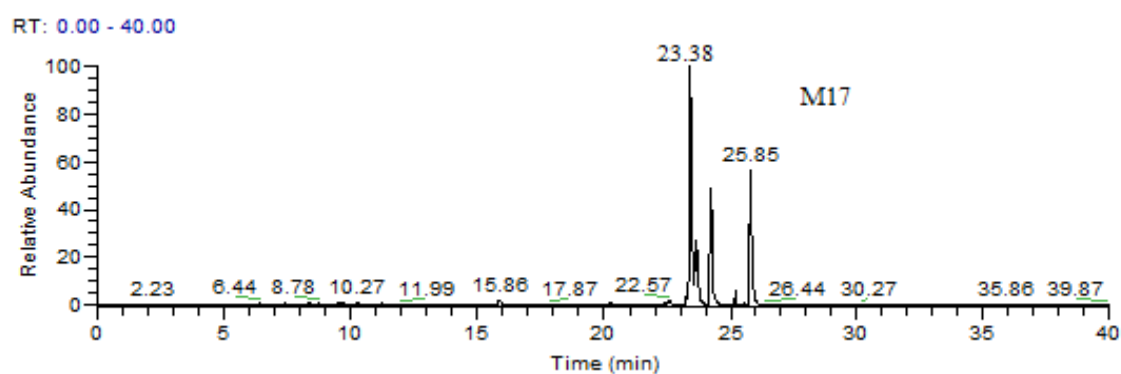

RT: 0.00 - 40.00

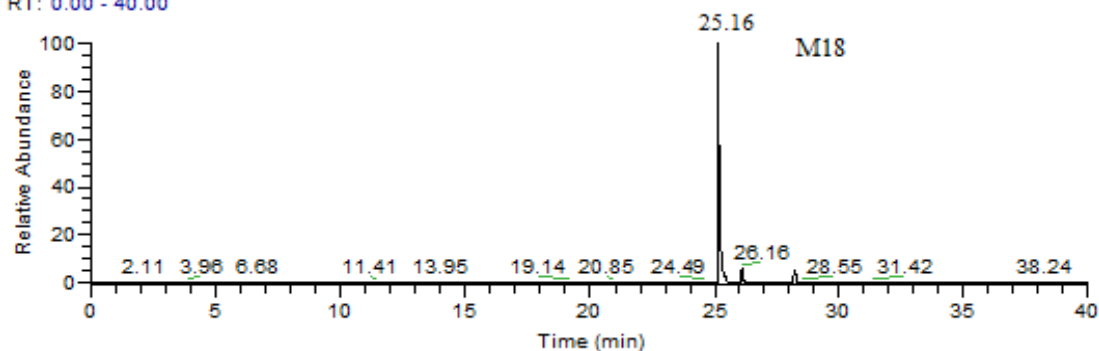

RT: 0.00 - 40.00

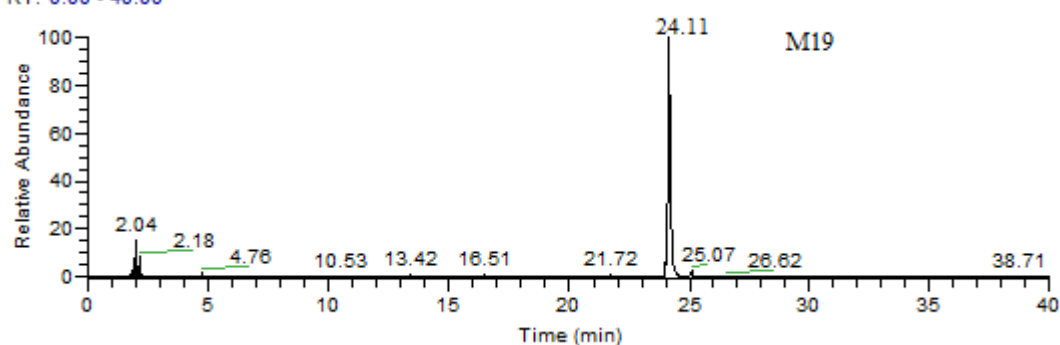

RT: 0.00 - 40.00

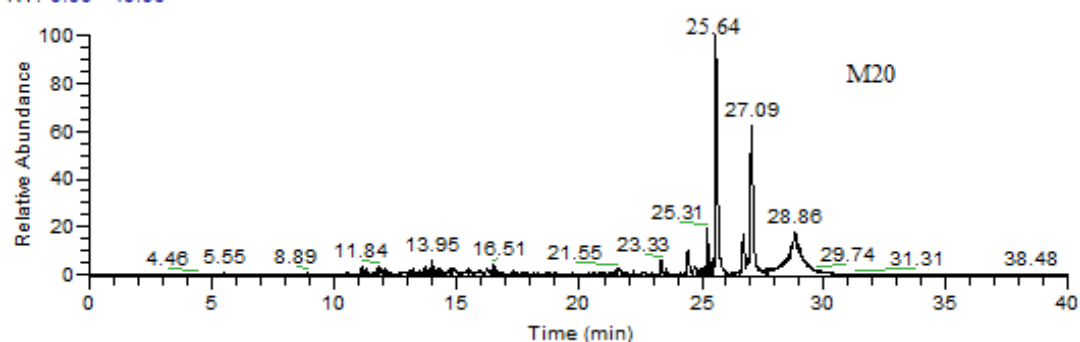

RT: 0.00 - 40.00

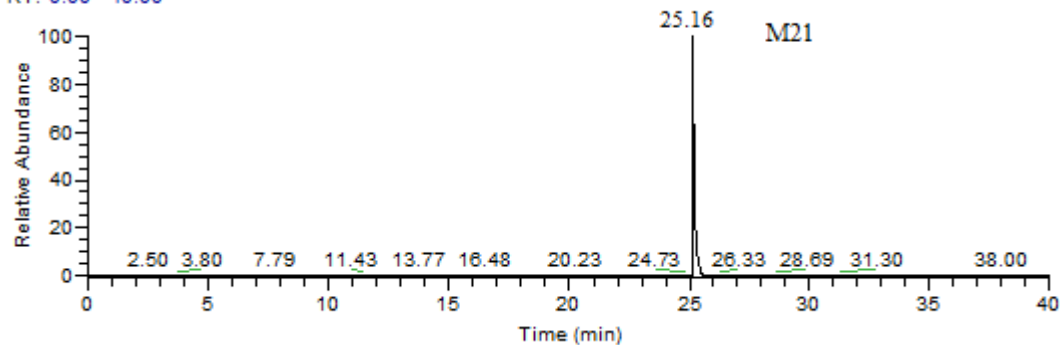

RT: 0.00 - 40.00

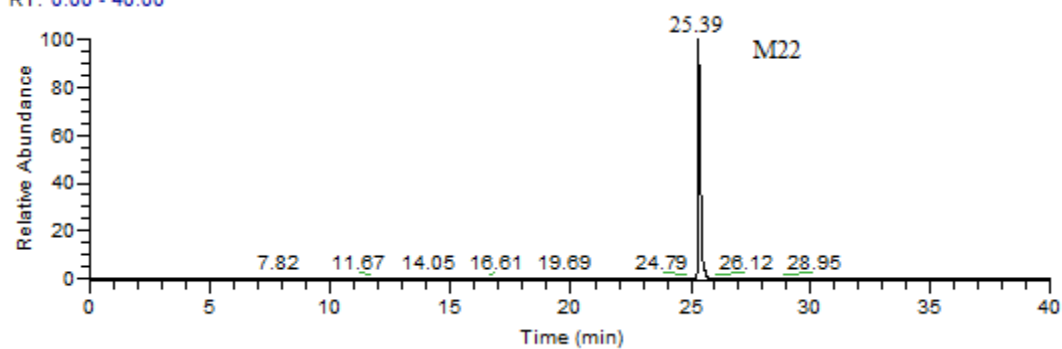

RT: 0.00 - 40.00

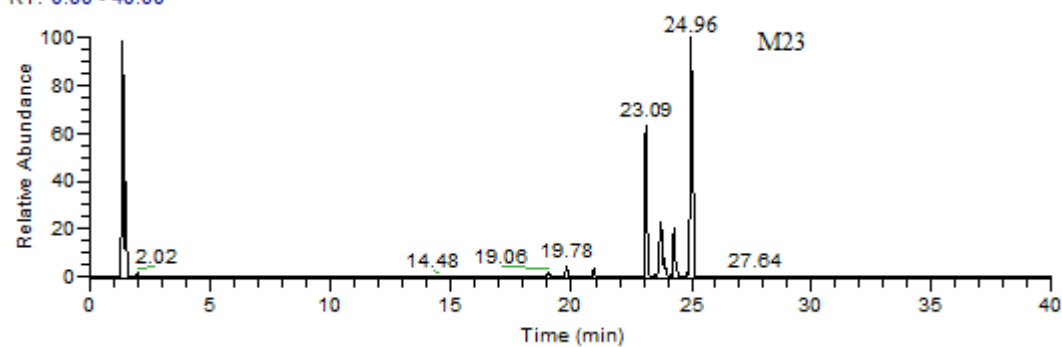

Supplement: Data Sheet 1 — The extracted ion chromatograms of metabolites and trepibutone. [file DataSheet_1.pdf]
